# Lethal gene drive selects inbreeding

**DOI:** 10.1093/emph/eow030

**Published:** 2016-11-08

**Authors:** James J Bull

**Affiliations:** 1 Department of Integrative Biology, University of Texas, Austin, TX, USA; 2 Institute of Cellular and Molecular Biology, University of Texas, Austin, TX, USA; 3 Center for Computational Biology and Bioinformatics, University of Texas, Austin, TX, USA

**Keywords:** genome engineering, selfish gene, population genetics, evolution, fitness

## Abstract

The use of ‘selfish’ gene drive systems to suppress or even extinguish populations has been proposed on theoretical grounds for almost half a century. Creating these genes has recently become possible with CRISPR technology. One seemingly feasible approach, originally proposed by Burt, is to create a homing endonuclease gene (HEG) that inserts into an essential gene, enabling heterozygote viability but causing homozygote lethality. With 100% segregation distortion in gametes, such genes can cause profound population suppression if resistance does not evolve. Here, population genetic models are used to consider the evolution of inbreeding (specifically selfing) as a possible response to a recessively lethal HEG with complete segregation distortion. Numerical analyses indicate a rich set of outcomes, but selfing often evolves in response to the HEG, with a corresponding partial restoration of mean fitness. Whether selfing does indeed evolve and its effect in restoring fitness depends heavily on the magnitude of inbreeding depression. Overall, these results point toward an underappreciated evolutionary response to block the harmful effects of a selfish gene. They raise the possibility that extreme population suppression may be resisted by mechanisms that are independent of the molecular basis of gene drive. At the same time, the evolution of inbreeding is not assured even if the genetic basis for inbreeding is present. As the models here strictly apply to hermaphrodites (plants), an important next step is to consider inbreeding in populations with separate sexes.

## Teaser

Gene drive has come of age with advances in genetic engineering. An unresolved question is whether gene drive systems will achieve population suppression or will instead succumb to resistance in the target population. Here is it shown that the evolution of inbreeding can be favored in response to a gene drive system, substantially reducing its efficacy.

## INTRODUCTION

The proposed use of selfish genes to suppress or extinguish populations is at least half a century old [1–3], but the feasibility of actually engineering selfish genes is new. There has thus been much excitement about the possibility of using these approaches to eradicate disease vectors, balanced by concerns about the possibility of unforeseen harm. Perhaps the most tangible approach is one outlined by [4], of creating a homing endonuclease gene (HEG) that inserts itself into an essential gene. Under the idealized assumptions of 100% segregation distortion in gametes of heterozygotes (germ line only), normal heterozygote viability and fertility but homozygote lethality, such a selfish gene is expected to evolve to such an extreme as to cause a 50% reduction in population fecundity if the segregation distortion is limited to one sex [5, 6]. A segregation distortion that operates in both sexes can evolve to fixation and death of all progeny, ensuring extinction [4, 6, 7]. A 50% reduction in population fecundity might seem to be so inconsequential as to eliminate it from further consideration. However, the 50% reduction applies to a single HEG, the combination of multiple HEGs at different locations in the genome (‘stacking’) has been proposed as a way of suppressing population fecundity to arbitrary levels [4].

The HEG need not work as completely as expected. Extreme levels of population suppression from the HEG are sensitive to even minor variations in parameter values [8, 9]. More importantly, an HEG that harms population fitness will select resistance mechanisms [10, 11]. Since HEGs target specific DNA sequences, the most obvious form of resistance to the HEG is a change in the target sequence so that the HEG will no longer duplicate itself in heterozygotes [4]. Resistance could also take the form of interfering with endonuclease expression or functionality. The problem of target sequence evolution has been countered with the suggestion of deploying multiple HEGs simultaneously [4], but other resistance mechanisms would not obviously be thwarted by that approach.

Here I address another possible evolutionary mechanism that may interfere with the spread and long term maintenance of a lethal HEG: evolution of inbreeding. It is appreciated that inbreeding reduces the population impact of ‘lethal’ HEGs and other gene drive systems [3, 4, 12, 13]. What is not clear is whether inbreeding is actually favored and how much it rescues mean fitness once a lethal HEG has invaded the population. Although a fixed level of inbreeding should reduce the incidence of the recessively lethal HEG, an allele that increases the level of inbreeding will itself suffer increased loss from any excess inviable progeny that it creates, perhaps selecting against inbreeding and even favoring increased outcrossing. It will in fact be shown here that inbreeding does evolve under some conditions, but the extent to which population fitness recovers depends heavily on the magnitude of inbreeding depression. Furthermore, the level of inbreeding that evolves is often not the level that would maximize population fitness were inbreeding imposed on the population.

The models analyzed here incorporate two major simplistic assumptions: the evolution of inbreeding is treated as the evolution of self-fertilization in an infinite population of simultaneous hermaphrodites with no spatial structure, and inbreeding depression is treated as a static quantity. Analysis of these models seems a reasonable first step in deciding whether the problem justifies inclusion of greater reality.

## THE MODELS

### Accommodating inbreeding as selfing

All models assume a population of diploid hermaphrodites capable of a mix of outcrossing and self-fertilization (selfing). Each individual produces a constant amount of sperm and of eggs. Eggs can be fertilized either by sperm chosen randomly from an outcross pool or by self sperm, from the individual who produced the ova.

The models assume that selfed offspring have a fitness lower than that of outcrossed offspring (*σ* < 1), with inbreeding depression parameter

δ=1−σ.

In this initial study, inbreeding depression is invariant throughout the evolutionary process. In real systems, inbreeding depression is often partially purged upon extended inbreeding, but allowing inbreeding depression to be static is a reasonable starting point and, if anything, provides a conservative measure of the vulnerability of gene drive systems to be suppressed by inbreeding (see Discussion section).

### Genotypes and phenotypes

The models assume two unlinked loci, each with two alleles (*A*/*a*, *D*/*d*). An individual’s level of selfing is controlled by its genotype at the *A*/*a* locus independently of the genotype at the other locus (Table 1). The *D*/*d* locus affects viability and experiences gametic drive. Specifically *D* is a recessive lethal that enjoys complete segregation distortion in heterozygotes—*Dd* produces all *D* gametes in spermatogenesis (and also in ovogenesis in some models). For most analyses, *Dd* is considered to be of normal viability and fertility (the drive does not operate in somatic tissues so those remain heterozygous and function normally), some analyses instead impart a slight fitness disadvantage to the heterozygote. The *DD* genotype of both sexes dies at conception, so viable genotypes at this locus are limited to *dd* and *Dd*.

**Table 1.** Genotype control of selfing rate

Genotype		Proportion ova selfed
aadd	aaDd	*s*_0_ = 0
Aadd	AaDd	*s*_1_
AAdd	AADd	*s*_2_

### Modifications of reproductive output

The net reproductive output of a genotype that produces selfed offspring or experiences zygote loss can be modeled in different ways, each of which may be observed in nature. For example, there are two extremes that span the possibilities for the impact of selfing on sperm contributed to the outcrossed pool [14, 15]. At one extreme, sperm contributed by an individual to the outcross pool may be reduced in proportion to the fraction of ova selfed by that individual (sperm are ‘discounted’). Alternatively, the outcross contribution may be unaffected (sperm not discounted) because few sperm are actually used for selfing compared with the huge number released for outcrossing. Both cases will be evaluated here.

In species with post-zygotic investment in offspring, reproductive compensation may also operate for lost zygotes, whereby the total number of viable offspring released from the maternal parent is larger than expected from the fraction of viable zygotes [15, 16]. Here, the models are limited to maternal offspring production in direct proportion to viable zygotes.

### Four models

The foregoing sections identified two important biological variations that affect the evolutionary dynamics:

(a) Drive operates just in male gamete production or in both male and female gamete production
(b)The contribution of sperm to the outcrossed pool is discounted in proportion to the fraction of ova selfed, or alternatively, selfing has no effect on the contribution to the outcross pool.

By considering these variables in all combinations, there are four models.

Interest is in whether selfing evolves specifically in response to the presence of *D* and the load from *DD* inviability. Appropriate analyses are thus limited to parameter values in which selfing would not evolve if *D* was absent. It is now well known that the evolution of selfing is sensitive to both the magnitude of inbreeding depression and whether selfed sperm are discounted [15, 17, 18]. When selfed sperm are not discounted from the outcross pool, there is an extra benefit to male function. Consequently, selfing is intrinsically beneficial at low values of inbreeding depression and is favored until inbreeding depression exceeds 0.5, the appropriate range for our problem is thus *δ* > 0.5. If instead selfed sperm are discounted, then selfing is favored only if inbred offspring are more fit than outcrossed offspring, so the appropriate range for our problem includes even small values of inbreeding depression, *δ* > 0.

The models will be analyzed for gene frequency evolution and mean fitness in the population (measured as viable offspring), equations are given in the Appendix. Applied interest in these evolutionary processes is primarily for population control and the potential for extinction [4, 19–21]. Yet, as has long been realized in applications of the sterile insect technique (used to suppress target pest populations), the impact of a particular intervention on adult population size depends heavily on ecology, which is often species-specific. Thus the analyses here omit any translation between mean fitness and population suppression.

## RESULTS

### Expectations

The evolution of gene drive has different consequences depending on whether drive operates in one sex (males, here) or both sexes. A drive allele with 100% distortion in sperm and ova of the same *Dd* heterozygote can evolve to the extreme that all offspring in the population are inviable *DD* [7]. This outcome ensures population extinction. Drive operating only in one sex can only evolve to the point that half the offspring are *DD*, half are Dd [5, 6] with a 50% reduction in population fitness (fecundity). A minimal expectation is thus that selfing should be favored for higher levels of inbreeding depression when drive operates in both sexes than when it operates just in one sex. For example, one might anticipate that selfing would not evolve in response to male-drive when inbreeding depression exceeds 0.5 (*σ* < 0.5) because mean fitness in the absence of selfing is 0.5.

### Evolutionary properties of single cases

When accounting for sex differences in segregation distortion, sperm discounting, inbreeding depression levels and initial conditions, there are many combinations of models to consider. To enable an easy comparison across this variety, a summary analysis of cases will be presented below. Before progressing to this summary, a few cases will be explored in detail. The first case will assume (i) gene drive in males only, (ii) no sperm discounting (no sperm lost to selfing), and (iii) inbreeding depression of *δ* = 0.51, just barely large enough to prevent the evolution of selfing in the absence of gene drive.

### Effect of selfing on mean fitness

**Figure 1.**
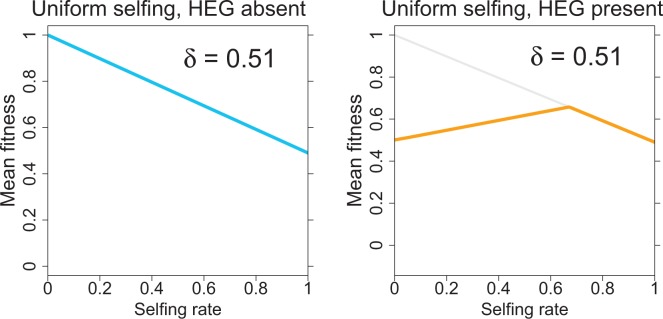
Fitness in a population with uniform selfing levels and inbreeding depression level *δ* = 0.51 (A) The blue line is mean fitness in the absence of the HEG (drive allele *D* is absent), the selfing rate is given on the lower axis. Mean fitness declines because higher levels of selfing impose inbreeding depression on more individuals. (B) Mean fitness when the HEG is present. The yellow line is equilibrium mean fitness when the drive allele *D* has evolved to equilibrium, under a uniform selfing rate given on the lower axis. The light gray line is the line shown in (A). *D* is lost for all points on the yellow curve that coincide with the gray line. The trials in (B) started with HEG heterozygotes at a frequency of 0.4

As a first step, mean fitness was calculated under the assumption that all individuals experienced the same level of selfing. In the absence of the HEG (allele *D*), mean fitness declines linearly with inbreeding, with a slope of −*δ* (blue line, Fig. 1A). With the HEG present and the system allowed to equilibrate, equilibrium fitness rises from 0.5 in the total absence of selfing to a maximum of 0.66 at a selfing level of 0.67 (yellow curve, Fig. 1B). This relationship appears linear up to this point. The HEG is maintained in populations with selfing rates less up to and including 0.67 but is lost in populations with levels of selfing of 0.68 and higher. With the HEG absent, the HEG-selfing mean fitness coincides with the inbreeding-fitness line and descends with yet higher imposed levels of selfing.

### Genetic variation in selfing: invasion and equilibrium fitness

The preceding analyses did not allow for the evolution of selfing, merely imposing a fixed level on the entire population. It might be inferred that, because mean fitness increases with selfing in the presence of the HEG (up to a point), selfing will evolve. But this evolution need not happen, or it may happen in unintuitive ways. An easy first step in evaluating evolution is to consider whether a selfing allele will invade a fully outcrossing population with the HEG at equilibrium. Equations for male drive, no sperm discounting (Appendix) were linearized for a rare allele *A* that increased selfing in a fully outcrossed population. Linearization assumed the equilibrium at which the HEG had gone to fixation with *aadd* absent, *aaDd* fixed.) For the genotype frequency vector (Aadd,AAdd,AaDd,AADd)′, the linearized transition matrix is

(1) $$\left(\begin{array}{cccc} \sigma s_{1}+\frac{1}{2} & 1 & 0 & 0\\ \frac{\sigma s_{1}}{2} & 2\sigma s_{2} & 0 & 0\\ \frac{3}{2}-s_{1} & 2\left(1-s_{2}\right)+1 & \frac{1}{2}\left(1-s_{1}\right)+\frac{\sigma s_{1}}{2}+\frac{1}{2} & 2-s_{2}\\ 0 & 0 & \frac{\sigma s_{1}}{4} & \sigma s_{2} \end{array}\right)$$

with leading eigenvalue (for the parameter range of interest)

(2) $$\begin{array}{c} \frac{1}{4}\left(\sqrt{4s_{1}^{2}\sigma^{2}+16s_{2}^{2}\sigma^{2}-16s_{1}s_{2}\sigma^{2}+12s_{1}\sigma -8s_{2}\sigma +1}\right)\\ +\frac{1}{4}\left(2\sigma s_{1}+4\sigma s_{2}+1\right)\;\;\;\;. \end{array}$$

**Figure 2.**
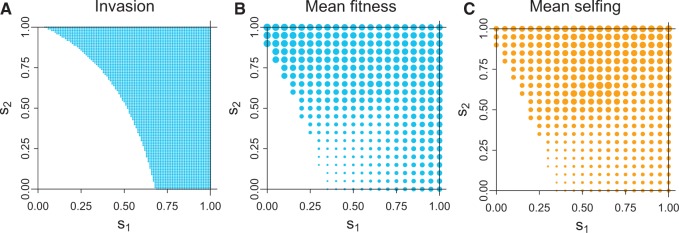
Invasion, equilibrium fitness and equilibrium inbreeding level as a function of genotype selfing rates (*s*_1_, *s*_2_) for the case of male drive, no sperm discounting with inbreeding depression parameter *δ* = 0.51 (as in Fig. 1) Panel (A) shows in blue the region of the parameter space for which invasion of the selfing allele occurs, from expression (2). Panel (B) gives equilibrium fitness attained by the allele, in which the diameter of the blue dot is proportional to the excess in fitness above 0.50. Maximal fitness in (B) is 0.67 at *s*_1_ = 0, *s*_2_ = 1. Panel (C) gives the average level of selfing at equilibrium, in which the diameter of the orange dot is proportional to the excess in fitness above 0.01. Maximal selfing in (C) is 0.65 at *s*_1_ = 0.65, *s*_2_ = 0.65. The analytically-derived invasion conditions of panel (A) are seen to occupy a slightly smaller range than the simulated conditions in (B). These discrepancies arise from the initial frequencies of allele *A* in the numerical trials being higher than is appropriate for the linearization assumptions. In (B), the region of invasion but low fitness (small points in the low, center part of the panel) is due to allelic constraints preventing the evolution of high selfing rates and thus preventing evolution of high mean fitness. Trials tested all combinations of *s*_1_ and *s*_2_ values in [0,1] incremented by 0.05. Initial frequencies in these runs were (0.93, 0.05, 0.01, 0.01, 0 and 0) for (*aadd*, *aaDd*, *Aadd*, *AaDd*, *AAdd* and *AADd*), chosen to represent rare selfing mutations present soon after the invasion of an HEG. Other initial conditions gave slightly different results regarding the evolution of selfing alleles near the boundary of the clear zone in (B)

Recall that σ=1−δ. A combination of analytical and numerical investigations of (2) reveals that selfing cannot evolve if *σ* does not exceed 0.25 (if *δ* ≥ 0.75). Regions of the (*s*_1_, *s*_2_) space where eigenvalue (2) exceeds 1.0 are plotted in blue in Fig. 2A for the case of *δ* = 0.51.

In addition to the invasion analysis, numerical analyses of the full equations were conducted, providing mean fitness and average selfing rates at equilibrium. Evolved mean fitness values are given in Fig. 2B, the diameter of each blue dot is proportional to the excess of mean fitness above the baseline 0.5. It is evident from both the invasion analysis and the equilibrium analysis that evolution of selfing requires that the *A* allele enacts high levels of selfing in either or both *Aa* and *AA* genotypes. Small levels of selfing do not invade, and as seen in Fig. 2B, the highest equilibrium fitnesses occur with *s*_2_ at or near 1.0. Selfing does not evolve in this system incrementally.

Fig. 2C gives the average level of selfing at equilibrium, with the diameter of the dot proportional to the selfing rate above 0.01. The highest levels of selfing occur in the middle of the parameter space, but these do not attain the highest fitness levels (although fitness levels in this region are not much below maximum).

It is easily inferred from Fig. 2B and C that (i) invasion of the selfing allele reduces the frequency of the drive allele *B* from its level with pure outbreeding, and (ii) invasion of the selfing allele does not usually lead to its fixation (because the average selfing rate in the population is often less than the value of *s*_2_). The former is easy to understand and plausibly follows from the analysis in Fig. 1B. The lack of fixation of selfing alleles presumably stems from a balance between the negative effect of inbreeding depression accompanying selfing and the benefit of selfing purging the drive allele, but an intuitive explanation for the specific outcome in individual cases seems out of reach.

To gain further insight to the interlocus dynamics, a correlation of the number of selfing alleles with the number of drive alleles within a zygote was calculated across all viable zygotes. Across many trials, the correlation at equilibrium was invariably negative, typically in the range of −0.2 to −0.3 when mean fitness was 0.6 or higher. This result matches the intuition offered above: the selfing allele experiences a diminished association with the drive allele presumably by purging it (see below for an exception).

**Figure 3.**
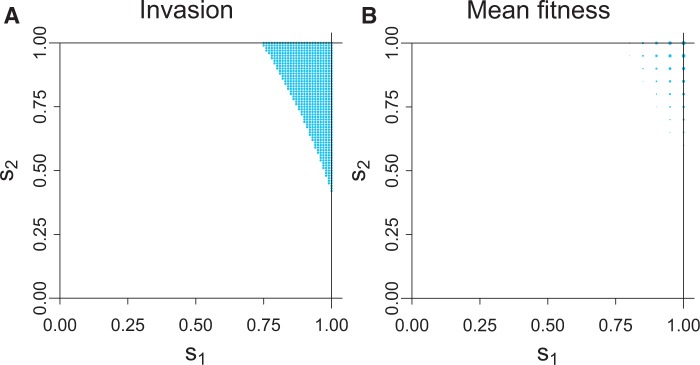
Invasion and equilibrium fitness as a function of genotype selfing rates (*s*_1_, *s*_2_) for the case of male drive, no sperm discounting with inbreeding depression parameter *δ* = 0.70 (as in Fig. 2 except for the different inbreeding depression parameter value) Invasion requires higher values of *s*_1_ and *s*_2_ than with *δ* = 0.51, and mean fitness is much lower when invasion does occur. (As in Fig. 2, the diameter of the blue dot is proportional to the excess in fitness above 0.50.) Maximal fitness in (B) is 0.56 at *s*_1_ = 1, *s*_2_ = 1. A plot of average selfing rates corresponding to that of Fig. 2C is not shown because it appears empty—the maximum observed selfing rate across the space is 0.028, too small to be visible as a point

For comparison, the same model was analyzed when the inbreeding depression parameter was set to *δ* = 0.7. Note that this value is near the expected boundary of 0.75, above which selfing cannot invade. The invasion region of the (*s*_1_, *s*_2_) space and equilibrium mean fitnesses are shown in Fig. 3. Average selfing rates are not shown because the values are too small to be visible using the same scale as in Fig. 2C.

A similar pattern was evident for male drive with sperm discounting (not shown). Invasion of the selfing allele *A* occurred for a much wider range of (*s*_1_, *s*_2_) values for *δ* = 0.01 than for *δ* = 0.45, again, values near ($s_{1}=0,\;s_{2}=0$) did not invade. However, for both models of 2-sex drive, invasion occurred for even the smallest selfing values tested ($s_{1}=0.01,\;s_{2}=0.01$).

The male-drive models have another interesting property regarding the evolution of selfing. At equilibrium with 100% segregation distortion and all individuals *Dd*, a mutant *A* allele will always arise in the *Dd* background, yielding a *AaDd* genotype. Henceforth, there will be no way for *A* to become paired with the *dd* genotype because all outcross and self pollen it receives are *D*−*A* is permanently trapped with *D*. Complete coupling of *A* with *D* ensures that the increased selfing imposes a load on *A* from the formation of *DD* homozygotes. Allele *A* cannot escape this load and is selected against relative to allele *a*. This outcome is, no doubt, a pathology of the strict parameter values that can be imposed in the model but would not likely apply in nature: The trapping of the selfing allele disappears if segregation distortion is less than complete, or if the starting population is not 100% *Dd*, whence *Aadd* is then formed and selfing can perpetuate it (and create *AAdd*). Nonetheless, the result reinforces intuition about the different processes affecting the selfing allele.

### Broad patterns in the joint evolution of selfing and gene drive

**Figure 4.**
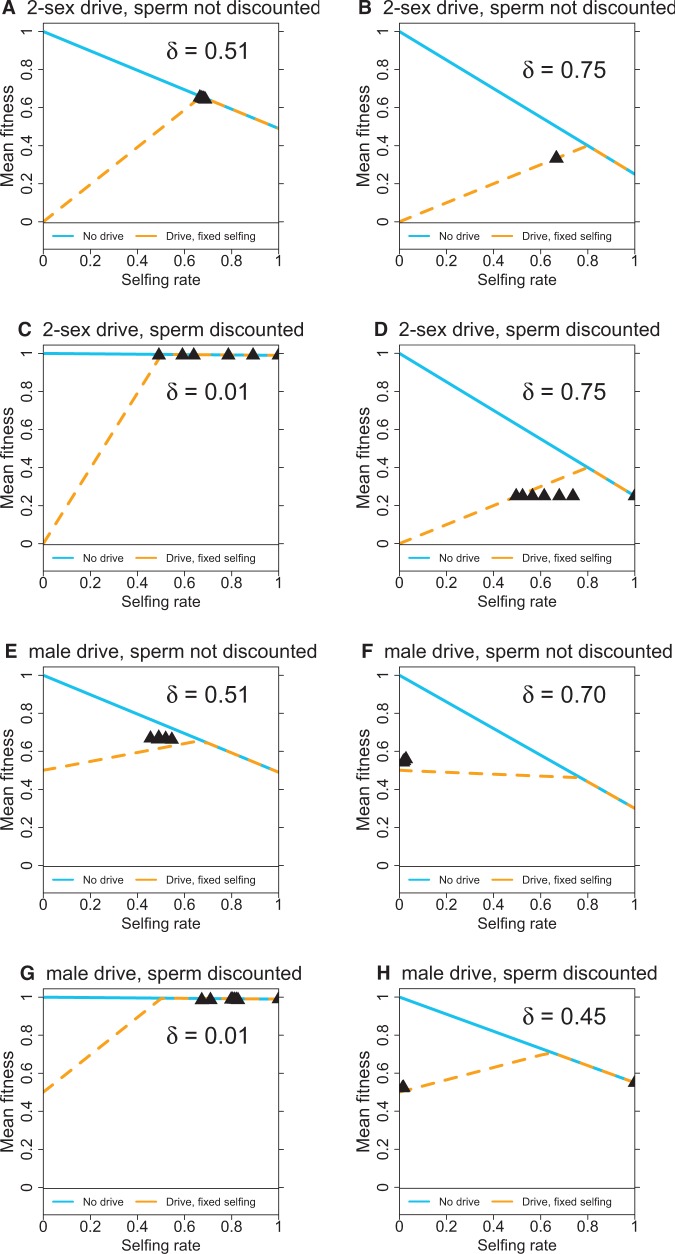
Outcomes of numerical trials for the four classes of models, each evaluated at two different inbreeding depression values (*δ*), as shown The blue and yellow curves represent mean fitnesses at equilibrium for a uniform selfing rate (with and without the HEG), as in Fig. 1. The black triangles represent equilibria when selfing was allowed to evolve in the presence of the HEG, showing outcomes at or near the highest mean fitness observed across hundreds of trials with different selfing genotypes (selfing rate in the graph is the population average selfing rate at equilibrium). For each *δ* value, four sets of initial genotype frequencies were analyzed at each of the 120 combinations of *s*1 and *s*2 values incremented by 0.1 across [0,1] (*s*1 = *s*2 = 0.0 was omitted). Initial genotype frequency sets were (0.49, 0.49, 0.01, 0.01), (0.93, 0.05, 0.01, 0.01), (0.05, 0.93, 0.01, 0.01), (0.25, 0.25, 0.25, 0.25) corresponding to (*aadd*, *aaDd*, *Aadd*, *AaDd*), initial frequencies of *AAdd* and *AADd* were 0. These initial conditions were chosen merely to span a range of initial frequencies. They had little effect on the outcome, except in the few cases that *D* was lost due to apparent floating point limitations (such cases are omitted from this figure, see the following section)

The four classes of models were analyzed for a total of four initial conditions across 120 different (*s*_1_, *s*_2_) values (Fig. 4). For each model, two different inbreeding depression values (*δ*) were chosen within the feasible range, panels (E) and (F) are from the analyses in Figs. 1, 2 and 3.

Each panel of Fig. 4 combines the two types of fitness curves of Fig. 1, again using blue for fitness in the absence of the HEG and (dashed) yellow when the HEG is present. The slopes and intercepts of the lines vary among panels, but the patterns are the same whereby the yellow curve rises to intersect the blue, and they then decline together at higher selfing levels. (In panel F, the yellow curve actually declines across the entire spectrum of selfing levels due to the high level of inbreeding depression.) For 2-sex drive, the yellow curves intersect the origin, reflecting the death of all individuals at equilibrium in a fully outcrossing population.

The numerical studies of Figs. 2 and 3 reveal a plethora of evolved mean fitness and average selfing rates across the spectrum of possible *s*_1_ and *s*_2_ selfing rates. Instead of presenting similar comprehensive data for each of the 6 other panels in Fig. 4, the panels show merely the highest mean fitness values and associated mean selfing levels obtained across the set of (*s*_1_, *s*_2_) values and initial conditions tested (black triangles). Where similarly high fitnesses were obtained across a range of average selfing rates, the triangles include those outcomes. The fitness values included were typically limited to within 0.02 of the observed maximum, with the goal of showing the span of average selfing rates giving rise to similarly high fitnesses.

There are several properties of these evolutions that defy intuition. The black triangles often do not coincide with maximum fitness on the yellow curve, and in a few cases they even lie moderately off the yellow curves. In Fig. 4E, the selfing alleles that attain maximal fitness are largely recessive, whereas those attaining maximal fitness in F are largely dominant, the two cases differ only in *δ*. Furthermore, it’s not even clear in Fig. F why selfing is favored, as mean fitness declines when selfing is imposed on the population (yellow curve). In some cases, the highest fitness spanned a considerable range in average selfing rates (e.g., Fig. 4C and D), whereas in other cases, they were limited to a tiny range [e.g., in both Fig. 4B and Fig. 4F, the three triangles fall on top of each other]. A more comprehensive analytical approach will be required to resolve these puzzles.

**Figure 5.**
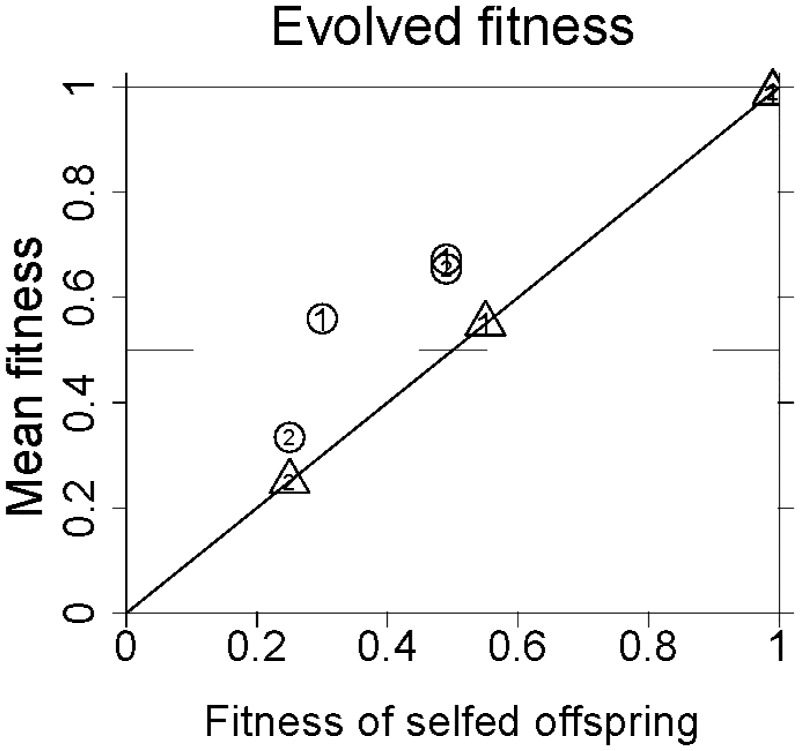
Maximal mean fitness observed in the different models (from Fig. 4) plotted as a function of the fitness of selfed offspring (σ=1−δ) Triangles indicate models with sperm discounting, circles are for models without sperm discounting. A ‘1’ or ‘2’ is shown within each symbol, indicating the number of sexes exhibiting drive of the *D* allele. The plot shows that fitness of selfed offspring correlates essentially perfectly with the maximum evolved fitness in the sperm discounting model. Evolved fitness is somewhat higher than *σ* when sperm are not discounted

A summary of the outcomes is given in Fig. 5. This is a plot of the maximum fitness observed versus the fitness of inbred offspring (*σ*) in each of the eight panels of Fig. 4. Each of the eight points is coded to indicate one-sex drive versus two-sex drive (‘1’ versus ‘2’) and whether sperm are discounted (triangles) or not (circles).

From the two preceding figures, several points are noteworthy.

- Given appropriate selfing rates for the *Aa* and *AA* genotypes, selfing evolved and increased mean fitness at least slightly above the fitness evolved with the HEG alone. Evolved fitness was equal to the fitness of selfed offspring (*σ*) for the sperm discounting models but somewhat above *σ* for the models with no sperm discounting.
- Mean fitness did not necessarily attain the maximum that could be obtained if selfing was imposed on the population.
- In the male-drive models, selfing never evolved if the fitness of selfed offspring (*σ*) was too low. Invasion analyses indicated that *σ* needed to exceed 0.5 in the sperm discounted model and 0.25 in models where sperm were not discounted.
- The drive allele (*D*) was lost only when a selfing rate of 1 could evolve (except for cases of apparent floating point error—see the following section).

Given that *DD* is lethal, the assumption that *Dd* is fully viable becomes questionable: fitnesses of recessive lethal heterozygotes are typically slightly below maximal [22]. The analyses of Fig. 4 were, therefore, conducted again but assigning a viability factor of 0.98 to all *Dd* genotypes. (A *Dd* produced by selfing had fitness 0.98*σ*.) Although quantitative effects of this fitness adjustment were observed, they were slight, and a parallel figure to that of 4 was effectively indistinguishable (not shown).

### A caution: the practical loss of drive for some initial conditions

In some models, certain initial conditions led to trajectories with vanishingly small frequencies of the *D* allele (e.g. 10^–50^ or less). When such low frequencies occur, the deterministic retention of *D* likely becomes irrelevant—it would be lost in any real population. The possibility of stochastic loss of *D* means that any implementation of a gene drive system should be analyzed stochastically for initial conditions that might be used.

**Figure 6.**
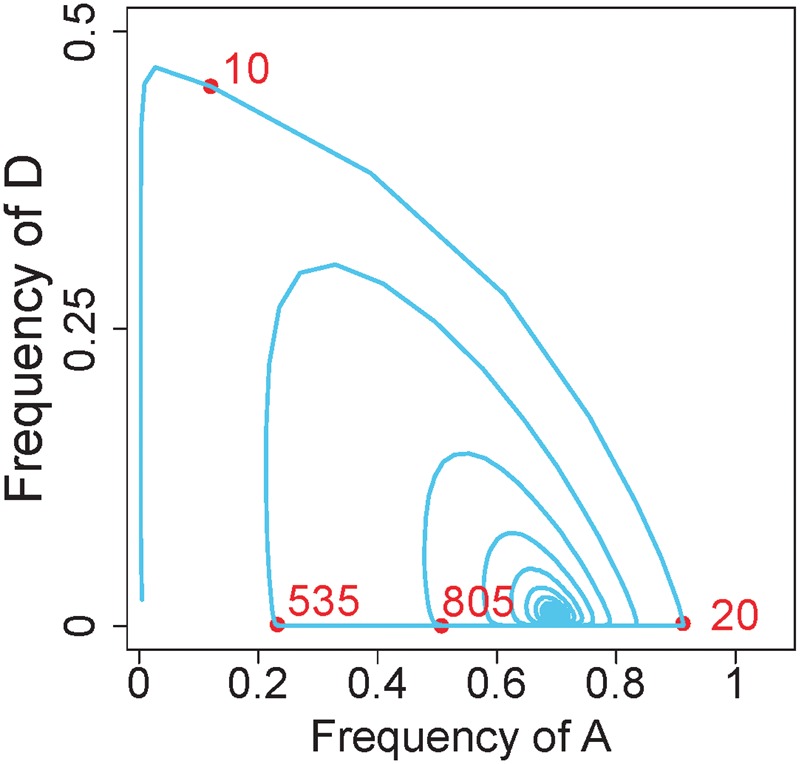
Numerical trajectory of allele frequencies (in the model of 2-sex drive, no sperm discounting) Red dots and associated numbers give the generation number at the point along the trajectory. Between generations 100 and 400, the frequency of allele *D* comes vanishingly close to extinction, less so in subsequent cycles. Initial frequencies of *aadd*, *aaDd*, *Aadd*, *AaDd*, *AAdd* and *AADd* were 0.95, 0.040, 0.005, 0.005 and 0,0, respectively. The model used for these dynamics was the one from Fig. 4A. $s_{1}=s_{2}=0.9,\delta =0.51$

For illustration, consider the model and *δ* value in Fig. 4A for *s*_1_ = *s*_2_ = 0.9. The presumed deterministic trajectory is shown in Fig. 6 for a specific set of initial frequencies. At first, *D* increases rapidly, in turn selecting increases in *A*. The rise in *A* and consequent increase in overall selfing drives *D* down to very low levels, less than 10^–30^ for over 250 generations. However, *A* declines during this period, allowing *D* to rebound, leading again to its rise and to further, damped cycles with *A*. The end point is an equilibrium with both *D* and *A*. *D* would be prone to extinction during these nadirs, and indeed, *D* was actually lost in some numerical trials (with slightly different initial frequencies than used in Fig. 6), until the floating point precision of the trial was increased. (That such losses were due to floating point error was independently confirmed by M. Edgington, personal communication.)

Anomalous loss of *D* was also seen in some numerical trials of other models. Again it seemed that the losses were not deterministic but instead resulted from inadequate floating point precision to maintain the infinitesimally low frequencies of *D*.

### Extended evolution of selfing

The models assumed a single, bi-allelic locus for selfing with an initial state of outcrossing (*s*_0_ = 0). It is possible that models allowing an extended evolution of selfing would lead to higher selfing rates with a higher mean fitness. Toward this end, further analysis was carried out of some trials in Fig. 4 in which the *A* allele had fixed and left the population with an intermediate level of selfing (*D* also present). The fixed *A* endpoint was then used to seed trials of the 2-locus model in which the evolved selfing rate was the baseline upon which further variation could act. Fig. 4B, D and E were evaluated: when a wide spectrum of selfing rates was tested against the new baseline, the opportunity for extended evolution often led to yet higher selfing rates and higher fitness above the starting state. Yet the extended evolution did not exceed the highest mean fitness found in the collective set of trails in Fig. 4 (black triangles), although extended evolution often achieved the same highest fitness, or nearly so.

Models restricted to two alleles at one selfing locus may also not provide full insight to long term evolutionary dynamics. A 3-locus model was created to investigate this possibility (two loci—*A*/*a* and *B*/*b*—affected selfing, the *D*/*d* locus encoded the drive).

The case of Fig. 4H was evaluated, as the mean fitness evolved under the 2-locus model was substantially below the maximum possible in the presence of the HEG. The initial conditions specified selfing rates of 0, 0.9 and 0.9 for the *aadd*, *Aadd* and *AAdd* genotypes, respectively, as led to the modest invasion of selfing in Fig. 4H. The *B* allele forced increases in the lowest level of selfing but maintained 0.9 as the highest level, thus evolution of *B* would have forced the baseline level of selfing above 0. None of those parameter combinations or the others tested resulted in an increase of *B*, suggesting that a multi-locus selfing system does not easily evolve to the level of selfing that would maximize mean fitness in the presence of the HEG.

Taken together, these results suggest that the trials for Fig. 4 captured the highest fitnesses capable of evolving under selfing (given a fixed value of inbreeding depression). There are clearly constraints on which selfing alleles are favored (e.g. Fig. 2), but the full suite of alleles considered may have captured the maximal fitness evolution via selfing.

## DISCUSSION

There is much justified excitement about the possibility of employing gene drive systems to limit wild populations of undesirable species [9, 12, 19, 20, 23]. In particular, homing endonuclease genes (HEGs) can now be developed that are recessive lethals but experience close to complete segregation distortion in heterozygotes [24]. Such HEGs can theoretically spread to fixation or to the point that the entire viable population is heterozygous [5–7], with a major reduction in mean fitness, possibly even extinction. Evolution of resistance to the HEG, or to its effects, becomes highly relevant in understanding the possible limitations of these engineered systems.

This study indicates that the final frequency of a recessive lethal enjoying complete segregation distortion can be reduced by the evolution of inbreeding. There is a consequent increase in mean fitness above that which evolves in the absence of selfing. One important result is that the fitness mitigation achieved by selfing is limited largely by the magnitude of inbreeding depression. In models assuming sperm discounting, selfing enabled mean fitness to avoid the low value expected from an uncontrolled HEG, but mean fitness at equilibrium was the same as that of a selfed offspring (σ=1−δ). Recovery was somewhat higher in the models with no sperm discounting (Fig. 5).

Although the deleterious population consequences of the HEG could be partly mitigated by the evolution of selfing, it was also true—in male-drive models—that selfing was favored only if the selfing allele enacted a sufficiently large degree of selfing. Alleles with low levels of selfing could not necessarily invade when alleles with high levels could. A conjecture is that an allele needs to attain high levels of selfing to purge the drive allele (which is a recessive lethal) and thereby escape the load that is otherwise associated with inbreeding.

The deterministic trajectory of the drive allele may traverse very low frequencies, values so low that allelic extinction would be expected in real populations. Indeed, some numerical trials appeared to lose the drive allele simply from inadequate floating point precision. Gene drive releases will thus require extensive analyses to identify initial conditions that are least prone to unwanted loss of the drive allele, although those trajectories will also depend on the presumably unknown genetics of inbreeding variants in the target population.

### Precedents in the evolution of mating systems

It has been long appreciated that suppressors of recessive lethal meiotic drive systems are strongly favored [10, 11]. However, the mechanisms of suppression may often be intricately tied to the molecular bases of the distorter [25]. The point of this paper is to suggest a type of suppression—inbreeding—whose origin and evolution is not sensitive to the molecular bases of the distortion. A resistance mechanism that is robust to the underlying mechanisms of distortion may thereby operate in many different systems.

The suggestion from this study is not without precedent, however. Evolution of inbreeding in response to a lethal HEG is one of now several examples of mating/genetic system evolution in response to a selfish allele (or alternatively, an altruistic allele). In some cases, it has merely been suggested that inbreeding limits the impact of a selfish element [e.g., 3, 4]. In one study, however, inbreeding was shown to evolve as a response to an evolving altruism [26]: the allele for increased inbreeding became coupled with the allele for altruism, with inbreeding reinforcing the benefit of altruism by increasing the number of altruists interacting in family units. A few studies have shown that the number of sires—polyandry or monogamy—evolves in response to the presence of a selfish element [27, 28] or in response to altruism [29], the number of sires affects relatedness within families and thus affects the competition between selfish and non-selfish alleles. In a system of yet higher complexity, Lande and Wilkinson [30] found that a sex-linked segregation distorter could be partially or wholly suppressed by female preference of a sex-linked male trait if that trait was tightly linked to the segregation distorter. Brandvain and Coop [31] found that segregation distorters in female meiosis can select changes in the female recombination rate.

## LIMITATIONS OF THE MODELS

### Other forms of inbreeding

With many proposed applications of gene drive systems to disease vectors and crop pests [4, 20, 21], the evolution of inbreeding as a possible ‘escape’ from a lethal HEG is especially relevant. The results here strictly apply to hermaphrodites (mostly plants), and even more narrowly to plants lacking self-incompatibility systems. There may well eventually be direct applications of these models in weed control, and the frequently observed evolution of selfing in plants [32] is a caution that such HEG implementations may have limited impact.

Yet the results of this study are also interesting for their implications to species with males and females, as in insects, where the most immediate uses of lethal HEGs and other genetic methods of control are entertained. For those species, selfing is not possible, so inbreeding would need to involve sib mating or some other localized mating structure. An obvious extension of the work here is thus to consider the evolution of inbreeding in those species. It might be anticipated that the results here will generalize to other forms of inbreeding, but further work is needed to go beyond mere speculation. Indeed, one potentially critical difference is that selfing more easily achieves high inbreeding coefficients than does sib mating. As suggested by R. Lande (personal communication), the fact that selection favored selfing alleles only if they enacted moderate to high rates of selfing (e.g. Figs. 2 and 3) may indicate that the evolution of sib mating will face greater constraints than does the evolution of selfing. Such a result would raise hope that the evolution of inbreeding is less likely to thwart an HEG in insects than in selfing species. However, the restrictions against evolution of low selfing rates were observed only for the male-drive models, so it is not clear whether the evolution sib mating would be constrained with 2-sex drive.

### Evolution of inbreeding depression

The models assumed that inbreeding depression (*δ*) was fixed throughout the evolutionary process. In contrast, inbreeding depression is known to evolve and can be at least partly overcome when inbreeding is imposed on formerly outcrossing populations [33]. In the results here, the magnitude of inbreeding depression played a critical role in invasion and ultimate recovery of population fitness (e.g. Fig. 5). Any purging of inbreeding depression would not necessarily be relevant to invasion conditions, but it would improve the recovery after invasion beyond that seen in models with fixed *δ*. An important extension of these models is thus to incorporate the evolution of inbreeding depression with the evolution of inbreeding.

Recent work points toward ways in which dynamic inbreeding depression might be accommodated. A consensus is emerging that inbreeding depression is due largely to deleterious mutations, but there are two important classes of deleterious mutations that contribute and have different consequences for purging: weakly deleterious mutations with large additive effects and strongly deleterious mutations such as lethals whose effects are largely recessive [14, 33, 34]. The weakly deleterious mutations are abundant, whereas the strongly deleterious mutations are much less common. In the short term, purging is chiefly from loss of the strongly deleterious class [14, 34]. A reasonable conjecture, therefore, is that results based on fixed inbreeding depression levels would accrue to inbreeding depression stemming from the weakly deleterious component, with the recessive lethal component being rapidly purged.

Another possible extension of the model is to expand the biological ramifications of inbreeding depression to include reproductive compensation for inviable zygotes. In species with post-zygotic parental investment in offspring, reproductive compensation boosts offspring number by replacing inviable genotypes with viable ones [16, 15]. The ramifications of this could be to facilitate the evolution of drive by supplementing *Dd* offspring to replace inviable *DD*.

### Implications for population suppression

The models analyzed here are of population genetics evolution. Yet interest in applying gene drive systems is often for population control—to limit numbers of adults. Unfortunately, across diverse ecological settings, there is no straightforward relationship between mean fitness and adult population size (the one exception being that a mean fitness of 0 ensures extinction).

Of particular relevance to population suppression by a recessive lethal HEG is that the lethality will typically operate at the zygotic stage. A reduction in zygotes will be especially prone to be overcome by many types of density-dependent regulation, whereby the number of adults is much less reduced than is the number of zygotes [35]. In the long history of work on the sterile insect technique—the goal of which is to suppress pest populations by artificially introducing sterile males or females—species-specific ecology often underlies the difference between success and failure [36–39]. It is thus to be expected that the impact of gene drive systems to suppress a target species will also be ecology dependent, unless of course it can destroy all progeny.

The models here merely indicate that selfing (and possibly other forms of inbreeding) will evolve if there is genetic variation for inbreeding and if inbreeding depression is not too severe. At least for mosquitoes—perhaps the most obvious target species for gene drive control efforts—there is hope that both constraints may obstruct the rapid evolution of inbreeding. There is a long history of inbreeding mosquitoes to establish laboratory stocks, they not uncommonly show strong inbreeding depression, and they also exhibit high heterozygosity despite inbreeding—suggestive of balanced lethals that will persist indefinitely as a load from inbreeding [40]. As to whether sib mating is even feasible in the absence of inbreeding depression, a survey of mosquito mating systems reveals that a swarming mating system is widespread in groups of disease-carrying mosquitoes, suggesting that they are neither predisposed to sib mating nor to adopting a life style that could easily convert to sib mating [41]. Pupal mating is known in some distantly related groups, however.

### Future

As noted above, an important direction motivated by the present study is extending the models to the organisms most likely to be targeted by lethal gene drive systems: mosquitoes and other insects, which have separate sexes. Such extensions would include sib mating instead of selfing, relevant ecology, and population structure. This level of modeling is obviously challenging, but models of gene drive spread with realistic ecology have already been developed [42–44], so adding the evolution of sib mating should be within reach.

The empirical feasibility of evolving high levels of inbreeding during assault by a recessive lethal HEG remains to be seen. We have essentially no field experience with gene drive systems. There is, however, over half a century of experience with various forms of sterile insect applications [45–47]. Sterile insect techniques almost universally rely on the release of lab-reared insects that, when mated with wild insects, cause death or sterility of the progeny [23]. The assault from sterilizing, lab-reared insects should also favor inbreeding as one of several mechanisms that avoid matings that produce sterile progeny. Yet resistance via assortative mating in wild populations subjected to the sterile insect technique has rarely been reported, despite many applications and successes (summarized in 13]. In this comparison, it may be important that the sterile insect technique relies on inundation of the wild population with lab-reared insects, the wild individuals becoming increasingly overwhelmed with sterility-inducing matings as the population declines. Gene drive systems may have the opposite effect, encouraging *de facto* consanguinity as the population density declines. Nonetheless, there are many reasons to be hopeful that gene drive systems will be able to achieve long-standing population control in at least some species.

## Appendix

The basic recursion equations are common to all models. Assuming discrete, non-overlapping generations:

(3) $$\begin{array}{c} XD\prime_{2,0}=S_{2,0}+M_{1,0}F_{1,0}\\ XD\prime_{2,1}=S_{2,1}+M_{1,1}F_{1,0}+M_{1,0}F_{1,1}\\ XD\prime_{0,0}=S_{0,0}+M_{0,0}F_{0,0}\\ XD\prime_{1,0}=S_{1,0}+M_{1,0}F_{0,0}+M_{0,0}F_{1,0}\\ XD\prime_{0,1}=S_{0,1}+M_{0,1}F_{0,0}+M_{0,0}F_{0,1}\\ XD\prime_{1,1}=S_{1,1}+M_{1,1}F_{0,0}+M_{1,0}F_{0,1}+M_{0,1}F_{1,0}+M_{0,0}F_{1,1}\\ X=D\prime_{2,0}+D\prime_{2,1}+D\prime_{0,0}+D\prime_{1,0}+D\prime_{0,1}+D\prime_{1,1}\;\;\;\;. \end{array}$$

Here, *D_i_*_,*j*_ represents a diploid adult, with *i A* alleles and *j D* alleles. Diploids may have 0, 1 or 2 *A* alleles but only 0 or 1 *D* alleles because *D* is a recessive lethal. *M_k_*_,*n*_ (*F_k_*_,*n*_) represents outcrossed male (female) gametes with *k A* alleles and *n D* alleles. *S_i_*_,*j*_ represents diploid offspring by selfing, with similar subscripting as for *D_i_*_,*j*_.

Recursion equations for the gametes and selfed offspring are specific to each model, as follows. It is assumed that all ova are fertilized, and sperm frequencies are normalized. Although the body of the paper restricts the analyses to complete drive, these equations use an unsubscripted *D* to represent the fraction of gametes of a *D* heterozygote carrying the *D* allele, facilitating the derivation. Selfing rates are *s*_0_ for *aa*, *s*_1_ for *Aa*, and *s*_2_ for *AA*, regardless of the other locus.

**Male-female drive, sperm discounted**

**Sperm**

(4) $$\begin{array}{c} TM_{1,0}=D_{2,1}(1-s_{2})(1-D)+D_{1,1}(1-s_{1})(1-D)/2\\ +D_{2,0}(1-s_{2})+D_{1,0}(1-s_{1})/2\\ TM_{0,0}=D_{1,1}(1-s_{1})(1-D)/2+D_{0,1}(1-s_{0})(1-D)\\ +D_{1,0}(1-s_{1})/2+D_{0,0}(1-s_{0})\\ TM_{1,1}=D_{2,1}(1-s_{2})D+D_{1,1}(1-s_{1})D/2\\ TM_{0,1}=D_{1,1}(1-s_{1})D/2+D_{0,1}(1-s_{0})D\\ T=M_{1,0}+M_{0,0}+M_{1,1}+M_{0,1} \end{array}$$

**Ova**

(5) $$\begin{array}{c} F_{1,0}=D_{2,1}(1-s_{2})(1-D)+D_{1,1}(1-s_{1})(1-D)/2\\ +D_{2,0}(1-s_{2})+D_{1,0}(1-s_{1})/2\\ F_{0,0}=D_{1,1}(1-s_{1})(1-D)/2+D_{0,1}(1-s_{0})(1-D)\\ +D_{1,0}(1-s_{1})/2+D_{0,0}(1-s_{0})\\ F_{1,1}=D_{2,1}(1-s_{2})D+D_{1,1}(1-s_{1})D/2\\ F_{0,1}=D_{1,1}(1-s_{1})D/2+D_{0,1}(1-s_{0})D \end{array}$$

**Selfed**

(6) $$\begin{array}{c} S_{0,0}=[D_{1,0}s_{1}/4+D_{0,0}s_{0}\\ +D_{1,1}s_{1}(1-D)(1-D)/4+D_{0,1}s_{0}(1-D)(1-D)]\sigma \\ S_{2,0}=[D_{2,0}s_{2}+D_{1,0}s_{1}/4+D_{2,1}s_{2}(1-D)(1-D)\\ +D_{1,1}s_{1}(1-D)(1-D)/4]\sigma \\ S_{1,0}=[D_{1,0}s_{1}/2+D_{1,1}s_{1}(1-D)(1-D)/2]\sigma \\ S_{2,1}=[2D_{2,1}s_{2}D(1-D)+D_{1,1}s_{1}D(1-D)/4]\sigma \\ S_{0,1}=[D_{1,1}s_{1}D(1-D)/2+2D_{0,1}s_{0}D(1-D)]\sigma \\ S_{1,1}=[D_{1,1}s_{1}D(1-D)]\sigma \end{array}$$

**Male-female drive, sperm not discounted**

**Sperm**

(7) $$\begin{array}{c} TM_{1,0}=D_{2,1}(1-D)+D_{1,1}(1-D)/2+D_{2,0}+D_{1,0}/2\\ TM_{0,0}=D_{1,1}(1-D)/2+D_{0,1}(1-D)\\ +D_{1,0}/2+D_{0,0}\\ TM_{1,1}=D_{2,1}D+D_{1,1}D/2\\ TM_{0,1}=D_{1,1}D/2+D_{0,1}D\\ T=M_{1,0}+M_{0,0}+M_{1,1}+M_{0,1} \end{array}$$

**Ova**

(8) $$\begin{array}{c} F_{1,0}=D_{2,1}(1-s_{2})(1-D)+D_{1,1}(1-s_{1})(1-D)/2\\ +D_{2,0}(1-s_{2})+D_{1,0}(1-s_{1})/2\\ F_{0,0}=D_{1,1}(1-s_{1})(1-D)/2+D_{0,1}(1-s_{0})(1-D)\\ +D_{1,0}(1-s_{1})/2+D_{0,0}(1-s_{0})\\ F_{1,1}=D_{2,1}(1-s_{2})D+D_{1,1}(1-s_{1})D/2\\ F_{0,1}=D_{1,1}(1-s_{1})D/2+D_{0,1}(1-s_{0})D \end{array}$$

**Selfed**

(9) $$\begin{array}{c} S_{0,0}=[D_{1,0}s_{1}/4+D_{0,0}s_{0}\\ +D_{1,1}s_{1}(1-D)(1-D)/4+D_{0,1}s_{0}(1-D)(1-D)]\sigma \\ S_{2,0}=[D_{2,0}s_{2}+D_{1,0}s_{1}/4+D_{2,1}s_{2}(1-D)(1-D)\\ +D_{1,1}s_{1}(1-D)(1-D)/4]\sigma \\ S_{1,0}=[D_{1,0}s_{1}/2+D_{1,1}s_{1}(1-D)(1-D)/2]\sigma \\ S_{2,1}=[2D_{2,1}s_{2}D(1-D)+D_{1,1}s_{1}D(1-D)/4]\sigma \\ S_{0,1}=[D_{1,1}s_{1}D(1-D)/2+2D_{0,1}s_{0}D(1-D)]\sigma \\ S_{1,1}=[D_{1,1}s_{1}D(1-D)]\sigma \end{array}$$

**Male drive, sperm not discounted**

**Sperm**

(10) $$\begin{array}{c} TM_{1,0}=D_{2,1}(1-D)+D_{1,1}(1-D)/2+D_{2,0}+D_{1,0}/2\\ TM_{0,0}=D_{1,1}(1-D)/2+D_{0,1}(1-D)+D_{1,0}/2+D_{0,0}\\ TM_{1,1}=D_{2,1}D+D_{1,1}D/2\\ TM_{0,1}=D_{1,1}D/2+D_{0,1}D\\ T=M_{1,0}+M_{0,0}+M_{1,1}+M_{0,1} \end{array}$$

**Ova**

(11) $$\begin{array}{c} F_{1,0}=D_{2,1}(1-s_{2})/2+D_{1,1}(1-s_{1})/4\\ +D_{2,0}(1-s_{2})+D_{1,0}(1-s_{1})/2\\ F_{0,0}=D_{1,1}(1-s_{1})/4+D_{0,1}(1-s_{0})/2\\ +D_{1,0}(1-s_{1})/2+D_{0,0}(1-s_{0})\\ F_{1,1}=D_{2,1}(1-s_{2})/2\pm D_{1,1}(1-s_{1})/4\\ F_{0,1}=D_{1,1}(1-s_{1})/4+D_{0,1}(1-s_{0})/2 \end{array}$$

**Selfed**

(12) $$\begin{array}{c} S_{0,0}=[D_{1,0}s_{1}/4+D_{0,0}s_{0}\\ +D_{1,1}s_{1}(1-D)/8+D_{0,1}s_{0}(1-D)/2]\sigma \\ S_{2,0}=[D_{2,0}s_{2}+D_{1,0}s_{1}/4\\ +D_{2,1}s_{2}(1-D)/2+D_{1,1}s_{1}(1-D)/8]\sigma \\ S_{1,0}=[D_{1,0}s_{1}/2+D_{1,1}s_{1}(1-D)/4]\sigma \\ S_{2,1}=[D_{2,1}s_{2}/2+D_{1,1}s_{1}/8]\sigma \\ S_{0,1}=[D_{1,1}s_{1}/8+D_{0,1}s_{0}/2]\sigma \\ S_{1,1}=[D_{1,1}s_{1}/4]\sigma \end{array}$$

**Male drive, sperm discounted**

**Sperm**

(13) $$\begin{array}{c} TM_{1,0}=D_{2,1}(1-s_{2})(1-D)+D_{1,1}(1-s_{1})(1-D)/2\\ +D_{2,0}(1-s_{2})+D_{1,0}(1-s_{1})/2\\ TM_{0,0}=D_{1,1}(1-s_{1})(1-D)/2+D_{0,1}(1-s_{0})(1-D)\\ +D_{1,0}(1-s_{1})/2+D_{0,0}(1-s_{0})\\ TM_{1,1}=D_{2,1}(1-s_{2})D+D_{1,1}(1-s_{1})D/2\\ TM_{0,1}=D_{1,1}(1-s_{1})D/2+D_{0,1}(1-s_{0})D\\ T=M_{1,0}+M_{0,0}+M_{1,1}+M_{0,1} \end{array}$$

**Ova**

(14) $$\begin{array}{c} F_{1,0}=D_{2,1}(1-s_{2})/2+D_{1,1}(1-s_{1})/4\\ +D_{2,0}(1-s_{2})+D_{1,0}(1-s_{1})/2\\ F_{0,0}=D_{1,1}(1-s_{1})/4+D_{0,1}(1-s_{0})/2\\ +D_{1,0}(1-s_{1})/2+D_{0,0}(1-s_{0})\\ F_{1,1}=D_{2,1}(1-s_{2})/2\pm D_{1,1}(1-s_{1})/4\\ F_{0,1}=D_{1,1}(1-s_{1})/4+D_{0,1}(1-s_{0})/2 \end{array}$$

**Selfed**

(15) $$\begin{array}{c} S_{0,0}=[D_{1,0}s_{1}/4+D_{0,0}s_{0}\\ +D_{1,1}s_{1}(1-D)/8+D_{0,1}s_{0}(1-D)/2]\sigma \\ S_{2,0}=[D_{2,0}s_{2}+D_{1,0}s_{1}/4+D_{2,1}s_{2}(1-D)/2\\ +D_{1,1}s_{1}(1-D)/8]\sigma \\ S_{1,0}=[D_{1,0}s_{1}/2+D_{1,1}s_{1}(1-D)/4]\sigma \\ S_{2,1}=[D_{2,1}s_{2}/2+D_{1,1}s_{1}/8]\sigma \\ S_{0,1}=[D_{1,1}s_{1}/8+D_{0,1}s_{0}/2]\sigma \\ S_{1,1}=[D_{1,1}s_{1}/4]\sigma \end{array}$$
